# Leaf Water Relations in Lime Trees Grown under Shade Netting and Open-Air

**DOI:** 10.3390/plants9040510

**Published:** 2020-04-15

**Authors:** Ana Belén Mira-García, Wenceslao Conejero, Juan Vera, María Carmen Ruiz-Sánchez

**Affiliations:** Irrigation Department, CEBAS-CSIC. P.O. Box 164, Campus de Espinardo, 30100 Murcia, Spain; abmira@cebas.csic.es (A.B.M.-G.); wenceslao@cebas.csic.es (W.C.); jvera@cebas.csic.es (J.V.)

**Keywords:** canopy temperature, drip irrigation, leaf water relations, lime trees, net house, thermal canopy index

## Abstract

Physiological plant water status indicators are useful for managing precision irrigation in regions with limited water resources. The aim of this work was to evaluate the effect of shade netting on the diurnal and seasonal variations of several plant water status indicators in young lime trees (*Citrus latifolia* Tan., cv. Bearss), grown at the CEBAS-CSIC experimental station in Murcia, Spain. Stem water potential (Ψ_stem_), leaf gas exchange (net photosynthesis (P_n_) and stomatal conductance (g_s_)), and canopy temperature (T_c_) were measured on representative days of winter and summer. The Ψ_stem_ daily pattern was quite similar in both seasons under both conditions. However, the circadian rhythm of leaf gas exchange was affected by shade conditions, especially in summer, when shaded leaves showed maximum g_s_ values for a longer time, allowing higher net photosynthesis (37%). Canopy temperature behaved similarly in both conditions, nevertheless, lower values were recorded in open-air than in shaded trees in the two seasons. The canopy-to-air temperature difference (T_c_ − T_a_), however, was lower in shaded trees during the daylight hours, indicating the higher degree of leaf cooling that was facilitated by high g_s_ values. The possibility of continuously recording T_c_ makes it (or the proposed canopy thermal index, CTI) a promising index for precise irrigation scheduling. Shade netting was seen to favour gas exchange, suggesting that it may be considered alternative to open-air for use in semi-arid areas threatened by climate change.

## 1. Introduction

Citrus is one of the most important crops in the world, with an annual production of 124 Mt, increasing by 10% over the last five years. Spain is the most important citrus fruit producer in Europe and the largest citrus exporter in the world (4.1 Mt) [1]. Citrus production is concentrated in the Comunidad Valenciana (3.1 Mt), Andalucía (2.2 Mt) and Region de Murcia (0.8 Mt) [2]. In Murcia, lemon has traditionally been the main citrus species (representing 64% of the province’s citrus production) [3], but in recent years others species such as lime have contributed to diversifying production, as growers seek a higher economic return than that offered by traditional local citrus varieties. Indeed, lime production in the region has increased by 25% in the last three years [4] and has become a good potential alternative for growers.

In forthcoming years, climate change is set to increase temperatures and hence evaporation [5], reducing water availability to an even greater extent, having a detrimental effect on fruit tree production worldwide. Under this scenario, especially in areas with limited water resources such as those in Mediterranean environments, the use of physiological water status indicators for precision irrigation management is considered a suitable way of adjusting the provided water to cover the needs of plants, thus saving significant amounts of water. Plant-based indicators rely on using the plant as a biosensor that integrates the soil and atmosphere water status, as well as measuring the physiological response to the water available in the soil. The most commonly used are the conventional, non-automated methods for measuring leaf or stem water potential, stomatal conductance or photosynthesis, and automated methods for measuring sap flow, canopy temperature, trunk diameter, or leaf turgor pressure [6]. Canopy temperature is one of the plant water status indicators that has recently become popular due to the development of infrared thermometry [6,7]. In the last few years, the use of canopy temperature for irrigation scheduling has been tested in several fruit tree crops, including cherry [8,9] avocado [10], almond [11], nectarine [12], peach [13] and also citrus trees such as lemon [14,15]. However, less information is available concerning lime.

Shading, which aims to protect crops against abiotic stresses such as high air temperature or excessive solar radiation, is regarded as a good cropping technique to cope with climatic change. This is likely due to the net increases light scattering, thus reducing radiation and creating an optimal microclimate for crop development [16]. As several studies have pointed out [17,18], shaded conditions affect agrometeorological variables, such as relative humidity, wind velocity and canopy and soil temperatures. Additionally, changes in the microclimate can modify physiological water status indicators. Many authors have pointed to the increased photosynthetic activity that occurs under reduced radiation, although it must be remembered that net-type affects this response [19,20,21]. The increase in CO_2_ uptake [22] and chlorophyll synthesis [20] induced by shading has been seen to increase tree growth and fruit yield, as reported for several citrus and other woody tree species [23,24,25]. Moreover, shade netting improves the quality of the fruit by boosting protection against biotic stress (diseases, insect pest) [26]. The ability of shade netting to improve water use efficiency, among other physiological traits, makes it a sound agricultural practice in areas with limited water sources.

For these reasons, the aim of this study was to evaluate the diurnal and seasonal variations of several plant water status indicators in lime trees, making discrete measurements of stem water potential (Ψ_stem_) and leaf gas exchange (stomatal conductance (g_s_) and net photosynthesis (P_n_)), and continuous measurements of canopy temperature (T_c_) in a Mediterranean environment of south-east Spain under two growing conditions: shade netting and open-air.

## 2. Results and Discussion

### 2.1. Meteorological Conditions

**Figure 1:**
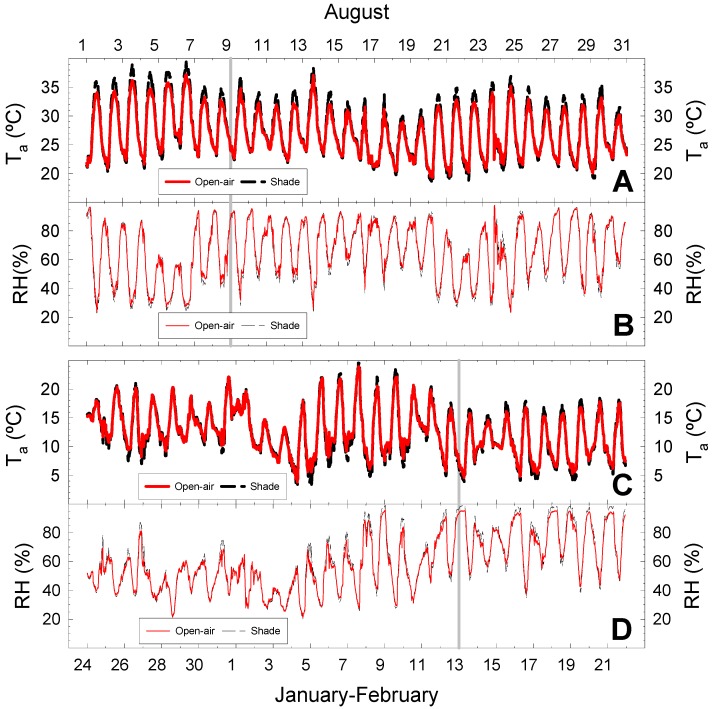
Diurnal pattern of air temperature (T_a_) and relative humidity (RH) during representative months of summer (August 2018; **A**,**B**) and winter (January–February 2019; **C**,**D**) in open-air (solid line) and under shade netting (dashed line). Vertical grey lines indicate days of measurements (9 August 2018 and 13 February 2019).

As is typical of Mediterranean climates, the meteorological conditions in the study area were more demanding in summer than in winter, with mean air temperature (T_a_) of 26 °C in summer (mean maximum and minimum of 32 and 21 °C, respectively) and 14 °C in winter (mean maximum and minimum of 16 and 9 °C, respectively). Air temperature and relative humidity (RH) were slightly different in the two cropping conditions in both seasons (Figure 1). In summer (August), the mean open-air T_a_ was lower (27.1 °C) and mean RH higher (65.5%) than in the shaded conditions (27.6 °C and 64.1%, respectively). Although only slight differences were noted in the mean values, the greatest differences were found at midday, coinciding with maximum T_a_ and minimum RH values (Figure 1A,B). Less pronounced differences between cropping conditions were noted during winter (February) with mean T_a_ = 12.1 and 12.0 °C, and mean RH = 61.5 and 62.8%, for open-air and shaded conditions, respectively. In this season, shaded conditions induced lower T_a_ than the open-air conditions during the night, but slightly higher values during daylight hours (Figure 1C,D).

**Figure 2:**
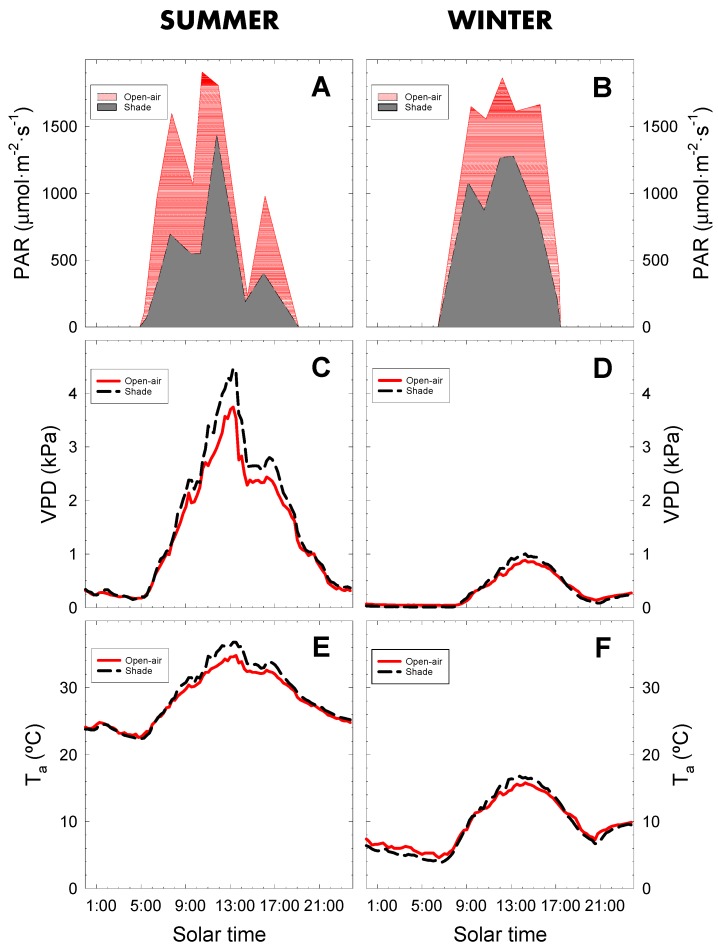
Diurnal course of incident photosynthetically active radiation (PAR), vapour pressure deficit (VPD) and air temperature (T_a_) on representative days of summer (9 August 2018; **A**,**C**,**E**) and winter (13 February 2019, **B**,**D**,**F**) in lime trees grown in open-air (solid line) and shaded (dashed line) conditions. Lines are the 15-min average values.

On the selected measurement days (9 August 2018 and 13 February 2019), T_a_ varied between 22.6 °C and 36.8 °C in summer and from 4.6 to 15.8 °C in winter, in open-air conditions (Figure 2E,F). The vapour pressure deficit (VPD) showed a similar pattern, with maximal values in summer (up to 4 kPa) and minimal values in winter (below 1 kPa) (Figure 2C,D). However, photosynthetically active radiation (PAR) was quite similar in both seasons, with maximum values (≈1900 µmol m^−2^ s^−1^), but with lower daylight hours in winter (Figure 2A,B).

Regarding the cropping conditions, the greatest differences in the agrometeorological variables were noted at midday, with higher T_a_ and VPD values in the shaded than in open-air conditions (Figure 2). In the summer, the maximum T_a_ was 2.0 °C lower in the open-air than under shade netting (Figure 2E), and 1.0 °C lower in winter (Figure 2F). Similar results were reported by Kührt et al. [27] in apple trees and Alaphilippe et al. [26] in pear trees, who observed that the air temperature in net conditions during daylight hours was 0.7 °C higher. However, the contrary was described by Blakey et al. [24] who observed that the air temperature was 2–3 °C lower in the shaded conditions to cultivate mango trees, while Gimeno et al. [28] observed a reduction up to 6 °C under the net shading cover provided for lemon trees. Blakey et al. [24] and Gimeno et al. [28] observed a reduction in the air temperature of 2–3 °C and 6 °C in mango and lemon trees, respectively under shaded conditions. Although all netting is intended for protecting trees and fruits from sunburn, mechanical injury from hail and wind, or other biotic/abiotic stresses, the contrasting results could arise from the different characteristics of the nets (net colour, shading factor, among others) [17,18].

As indicated above (Figure 1), RH values at midday were slightly lower in shade (mean 30%) than in open-air (33%) conditions. Similarly, Hunsche et al. [29] and Solomakhin and Blanke [30] observed a reduction (2–5%) in RH under netting. As a result of the higher T_a_ and lower RH in shade conditions, VPD values were higher than in the open-air conditions, especially in summer when the atmospheric conditions were more demanding (Figure 2C,D). This fact could be mediated by the semi-permeable characteristics of the net and the architecture of the net-house (see Materials and Methods) that caused less air renewal and, therefore, reduced the advective effect inside the net house.

During night-time hours, T_a_ values were similar in both cropping conditions in summer (Figure 1A and Figure 2E), although there were some differences in the winter, when T_a_ was lower (≈1.5 °C) in shaded than in open-air conditions (Figure 1C and Figure 2F). A slight decrease in the air temperature (0.4 °C) during the night hours was noted under anti-hail black netting [27]. However, no differences were noted in VPD values between cropping conditions at this time in either season (Figure 2C,D).

PAR values were significantly lower inside the net house than in open-air conditions. The area under the PAR curves (Figure 2A,B) shows that net shading reduced the level of radiation by 53% and 30% in summer and winter, respectively. García-Sánchez et al. [31] observed a substantial (≈50%) reduction in PAR values in shaded lemon trees, compared with the 39% reduction observed in orange trees by Zhou et al. [21]. This reduction in the incident PAR in the shade would be due to the light scattering effect of the net, which disperses the light and only allows part of incident radiation through.

### 2.2. Stem Water Potential

**Figure 3:**
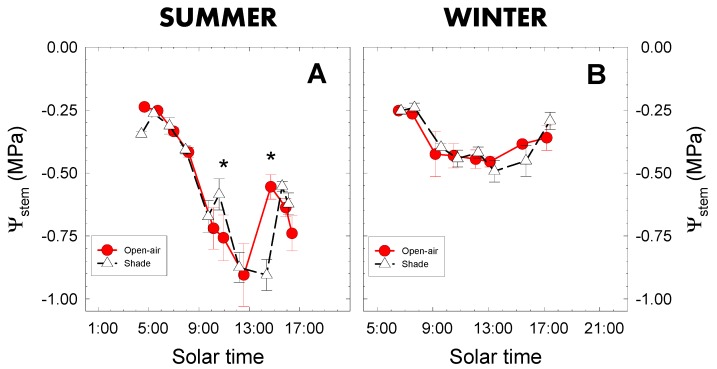
Diurnal course of stem water potential (Ψ_stem_) on representative days of summer (9 August 2018; **A**) and winter (13 February 2019; **B**) in lime trees grown in open-air (closed symbols) and shaded (open symbols) conditions. Each point is the average of four replications. Vertical bars represent ± standard error (SE). Asterisks indicate statistically significant differences between cropping conditions at * *p* ≤ 0.05.

During the experimental period, the trees were not affected by limiting soil water conditions, as the stem water potential (Ψ_stem_) confirmed, with values > −0.9 MPa (Figure 3A,B), indicating that the plant did not suffer water stress [15,32,33]. As in most cultivated plants, Ψ_stem_ in both cropping conditions showed a circadian pattern characterized by higher values at the beginning of the day, after which the values gradually decreased as evaporative demand increased, reaching a minimum at around midday [15,19,34,35]. After midday, as a consequence of a decrease in the evaporative demand, the Ψ_stem_ gradually increased until the end of the day (Figure 3A,B).

**Figure 4:**
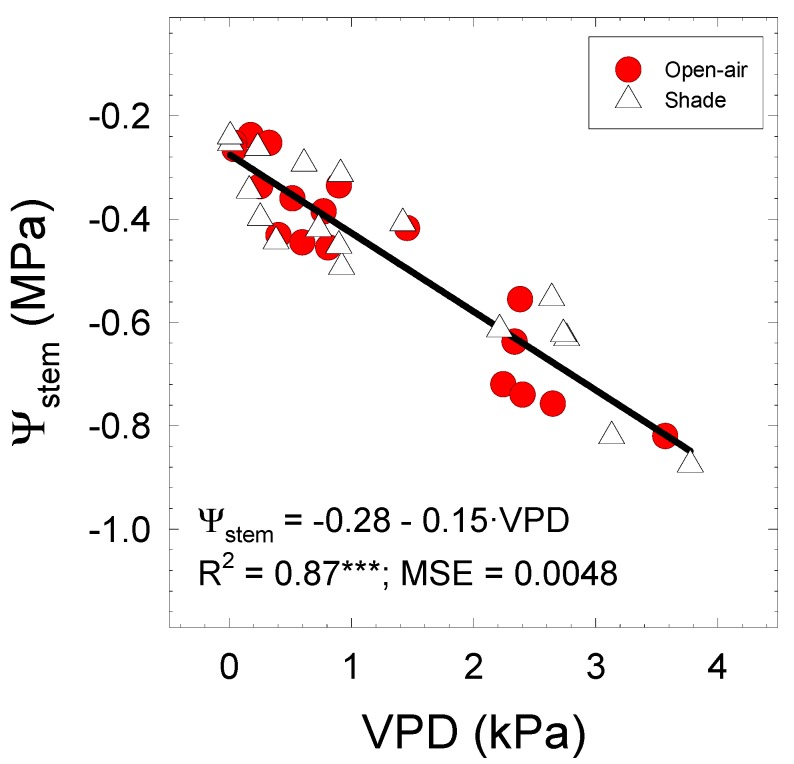
Relationship between daily stem water potential (Ψ_stem_) and vapour pressure deficit (VPD) values in lime trees grown in open-air (closed symbols) and shaded (open symbols) conditions on the two measurement days. Each point is the average of four replications. *** *p* ≤ 0.001. MSE = mean squared error.

In summer Ψ_stem_ values fluctuated between −0.25 and −1.0 MPa, while in winter between −0.25 and −0.5 MPa. In winter, no statistically significant differences were observed between cropping conditions. However, in summer, significant differences were observed around 11:00 and 15:00 h solar time when shaded trees showed lower Ψ_stem_ values than open-air trees (Figure 3A,B). The more negative Ψ_stem_ values coincided with the higher VPD values (Figure 2C,D), likely due to the linking between Ψ_stem_ and evaporative demand of the atmosphere. In our experimental conditions, Ψ_stem_ was closely related to VPD, as demonstrated by the strong correlation between VPD and Ψ_stem_ (Figure 4). Higher determination coefficient values (R^2^) were found when open-air and shade net conditions were considered separately but the covariance analysis indicated no differences between the regression coefficients. Similar relationships have been observed in studies made in lemon [15], peach [36], apricot [35], almond [37] and avocado [10], highlighting the role of the atmosphere as a driving force for the water transference processes, and emphasizing the suitability of Ψ_stem_ as a robust plant water status indicator [38,39,40].

### 2.3. Gas Exchange

**Figure 5:**
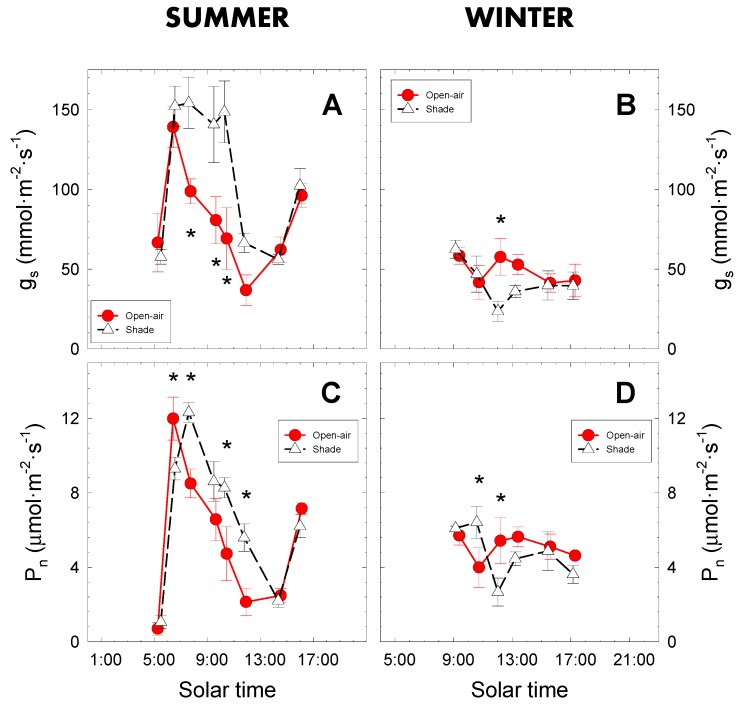
Diurnal course of stomatal conductance (g_s_) and net photosynthesis (P_n_) on representative days of summer (9 August 2018; **A**,**C**) and winter (13 February 2019; **B**,**D**) in lime trees grown in open-air (closed symbols) and shaded (open symbols) conditions. Each point is the average of four replications. Vertical bars represent ± standard error (SE). Asterisks indicate statistically significant differences between cropping conditions at * *p* ≤ 0.05.

The diurnal courses of leaf gas exchange (stomatal conductance (g_s_) and net photosynthesis (P_n_)) pointed to maximal stomatal opening during the early morning hours and minimum values at midday [41] in both conditions, as illustrated in Figure 5. In summer, g_s_ in open-air grown trees increased rapidly and then decreased rapidly, whereas shaded trees maintained significantly higher stomatal conductance values until midday (Figure 5A), resulting in a 54% higher accumulated daily g_s_ compared with open-air trees. In the same way, an increase of stomatal conductance (more than 50%) was observed in several apple tree cultivars grown under black shade net compared with those grown in open-air conditions [42].

A similar daily pattern was observed for net photosynthesis in summer, with significantly higher mean daily values (37%) in shaded trees, although a delay in reaching maximum P_n_ values was noted with respect to open-air trees (Figure 5C). These higher leaf gas exchange levels under shade conditions could be due to the lime trees being exposed to lower incident PAR radiation (Figure 2A), and such non-saturated conditions would allow the stomata to remain open longer (Figure 5A). The higher stomatal opening would improve CO_2_ uptake [22], increasing the photosynthetic capacity of shaded trees, as several studies have reported [16,20,43]. It should be mentioned that a cloudy event (around 15:00 solar time) caused an abrupt decrease in PAR (Figure 2A) and VPD (Figure 2C) values. This unexpected condition did not consistently affect the leaf gas exchange parameters (Figure 5A,C), which were following the circadian rhythm of stomatal cycling (Figure 5A) as has been similarly reported in citrus species [34,44,45,46].

In winter, the less demanding climatic conditions led to lower gas exchange values, which behaved similarly in both cropping conditions, with only slight significant differences before midday when trees cultivated in the open-air had slightly higher values than the trees grown in shaded conditions (Figure 5B,D).

The climatic conditions clearly influenced gas exchange [44], but may also have been affected by the feedback effect of the fruits on leaf photosynthesis [47], with higher P_n_ and g_s_ values recorded in August than in February (Figure 5). The presence of fruits at their maximum growth rates just before harvesting (at the end of August 2019) would have been responsible for this behaviour, as found in *Citrus* [15] and *Prunus* sp. [35,48].

The daily gas exchange dynamics in both cropping conditions showed maximum stomata opening and higher net photosynthesis values early in the morning, as seen in other studies [33], confirming that leaf gas exchange in lime trees should be measured at this time of the day for comparative purposes. Moreover, based on the higher correlations found with maximum daily g_s_ values [15], Ψ_stem_ should be measured at midday.

### 2.4. Canopy Temperature

**Figure 6:**
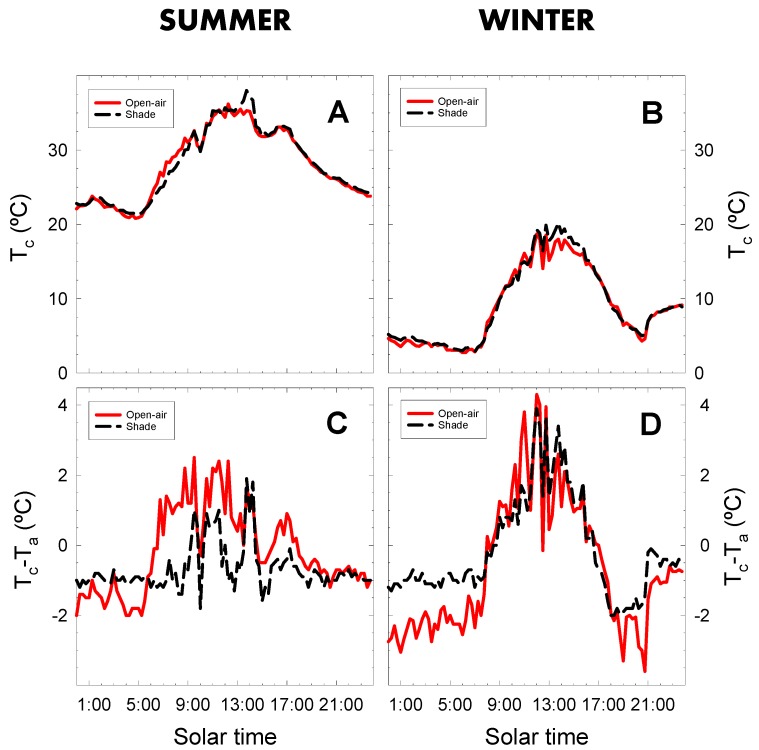
Diurnal course of canopy temperature (T_c_) and canopy-to-air temperature difference (T_c_ − T_a_) on representative days of summer (9 August 2018; **A**,**C**) and winter (13 February 2019; **B**,**D**) in lime trees grown in open-air (solid line) and shaded (dashed line) conditions. Lines are the 15-min average values of two replications.

The development of infrared thermometry techniques has made it possible to measure canopy temperature remotely, without any physical contact with the plant. The continuous canopy temperature (T_c_) records for the lime trees showed values varying from 20 to 40 °C in summer, and from 3 to 20 °C in winter (Figure 6A,B). The minimum T_c_ values were recorded at the beginning and the end of the day, and maximum T_c_ values around midday. In both seasons, the canopy temperature from dawn to dusk was very similar in both cropping conditions. However at midday, when the meteorological conditions were more demanding (Figure 2), plants grown in shade conditions had a higher T_c_ (2.70 and 2.65 °C, in summer and winter, respectively) than in those grown in open-air conditions (Figure 6A,B). Blackey et al. [24] also observed an increase of 10% in maximum T_c_ in avocado as a result of shade netting. However, in orange trees [21], canopy temperature was similar in non-netted (35.4 °C) and shaded (35.3 °C) trees. The slight increase in the T_c_ of shaded lime trees (Figure 6A,B) could have been due to the high T_a_ and low RH values measured inside the net house at midday in both seasons (Figure 1). However, it should be noted that the microclimate of the leaf boundary layer was slightly different from the measurements of the climatic sensors (Figure 2C,D), with lower VPD_leaf_ values in shaded than in open-air conditions, as measured by the LICOR, LI-6400.

Shaded lime trees, however, showed lower values for the canopy-to-air temperature difference (T_c_ − T_a_) than the trees grown in open-air conditions, particularly in summer, when T_c_ − T_a_ was negative during the greatest part of daylight hours (Figure 6C). This reduction in T_c_ − T_a_ led to a higher degree of leaf cooling in shaded trees as a consequence of the increase in stomatal conductance in the shade net conditions (Figure 5A). In winter, T_c_ − T_a_ values were quite similar in the two cropping conditions during daylight hours, while lower values were noted in open-air than in shaded trees during the night (Figure 6D).

Canopy temperature is recognized as a sound plant water status indicator [6,7,49] and is affected by the prevailing environmental conditions. Rather than absolute T_c_ values, the difference between the canopy and air temperature has been proposed as a sound indicator of plant water status. High T_c_ − T_a_ values indicating water stress and low values well-irrigated conditions [50,51]. The higher T_c_ − T_a_ values measured in lime trees grown in the open-air than in shaded trees (Figure 6C) would result from stomatal closure (Figure 5A).

**Figure 7:**
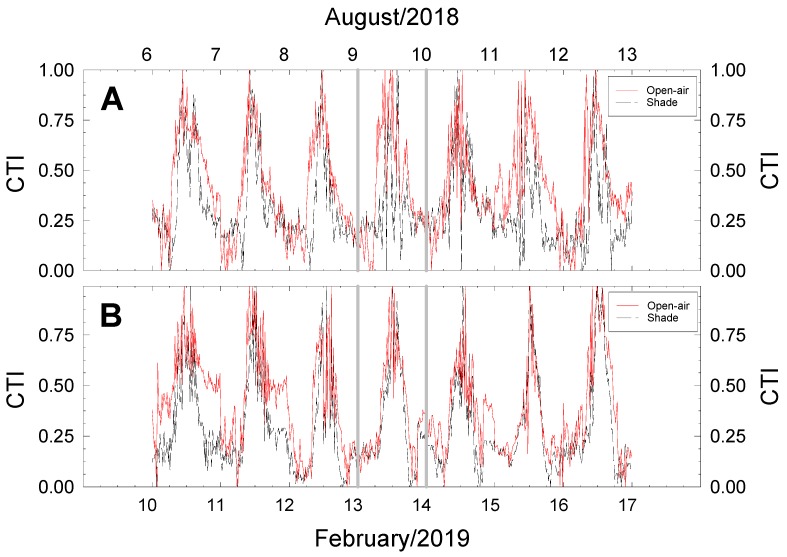
Canopy thermal index (CTI) for representative weeks of summer (6–13 August 2018; **A**) and winter (10–17 February 2019; **B**) in lime trees grown in open-air (solid line) and shaded (dashed line) conditions. Vertical grey lines indicated the measurement days (9 August 2018 and 13 February 2019). Lines are the 15-min average values of two replications.

The proposed canopy thermal index (CTI) based on the normalised T_c_ − T_a_ values (Equation (1)) under the two cropping conditions during one week of summer and winter is presented in Figure 7. The CTI pointed to lower values in shaded than in open-air grown trees during most of the day (Figure 7). When the respective measurement days are examined, a close relationship can be seen between CTI and plant water status—the higher the CTI the lower the Ψ_stem_ -, as observed in predawn on 9 August (Figure 3A) and during the early afternoon of 13 February (Figure 3B). Additionally, the higher CTI values would explain the higher T_c_ recorded in shaded plants in both seasons (Figure 6A,B).

The integrated daily CTI values (∑_24h_CTI) were 7.24 and 10.00 for shaded and open-air trees, respectively, on the above mentioned day in summer, and 7.56 and 8.93 in winter. This behaviour was maintained when the weekly data in Figure 7 were analysed, statistically significant differences between cropping conditions being evident: an average ∑_24h_CTI of 7.57 ± 0.44 and 10.02 ± 0.39 for shaded and open-air trees in August, and 7.04 ± 0.36 and 9.49 ± 0.75 in February, respectively.

Although more studies are required, the fact that low CTI values were associated to better plant water status (lower Ψ_stem_) empowers the sensor-based of continuous T_c_ measurements as a sound water status indicator in lime trees, thus canopy temperature, in terms of CTI, is proposed as a tool for irrigation management in areas with scarce water resources.

## 3. Materials and Methods

### 3.1. Plant Material and Experimental Conditions

The experiment was carried out during the 2018–2019 growing season at the experimental field station of CEBAS-CSIC in Murcia, Spain (38°06′31″ N, 1°02′14″ W, 110 m a.s.l.). The plant material comprised three-year-old lime trees (*Citrus latifolia* Tan., cv. Bearss) grafted on *Citrus macrophylla* L. rootstock. The soil of the 0–0.5 m layer was stony, highly calcareous (45% calcium carbonate), with a clay loam texture and low organic matter content. The average bulk density was 1.43 g cm^−3^. The soil water content at field capacity and permanent wilting point were 0.29 and 0.14 m^3^ m^−3^, respectively.

Trees were planted 5 m apart in raised rows (2 m wide and 0.4 m high), which were 6 m apart. The trees were irrigated with a double drip lateral line separated 1 m, with four pressure-compensated emitters, of 4 L h^−1^, located 0.7 m from the tree trunk. The trees were irrigated to fulfil plant water requirements throughout the experiment. The irrigation scheduling was automated with soil management that allowed depletion (MAD) of 10% [52]. The soil water content was monitored at a depth of 0.8 m by means of multi-depth capacitance probes (EnviroPro^®^, Entelechy Pty. Ltd., Golden Grove, Australia) and the measurements served as feedback for irrigation control by acting on electrovalves that formed part of a telemetry system. The values were read every 5 min and the averages were recorded every 15 min. The radio-transmission units sent data to a gateway connected to addVANTAGE web server software (ADCON Telemetry, Klosterneuburg, Austria) for data processing and visualisation. Eight soil probes were calibrated and installed 0.1 m from the selected emitter of representative trees (four for each cropping condition).

### 3.2. Cropping Conditions

**Figure 8:**
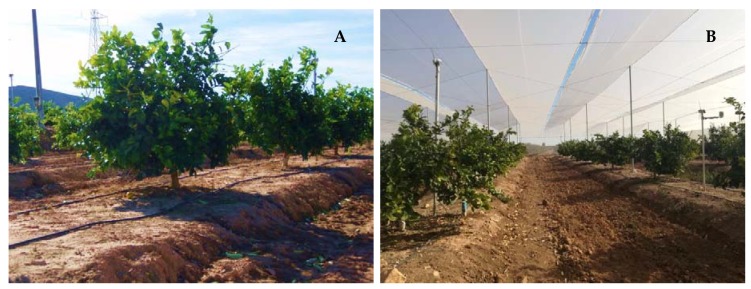
General view of lime trees (*Citrus latifolia* Tan., cv. Bearss) grown in open-air (**A**) and shade net house (**B**) conditions.

The trees were subjected to two cropping conditions: open-air (Figure 8A) and shade netting (Figure 8B), each consisting of 60 trees. Both cases received the same amount of irrigation water. The shade netting formed a net house 4.5 m high, with a zig-zag roof (Figure 8B). The HDPE white agricultural net used had a 76% light transmission and 6 × 9 strands cm^−2^ (CYLNET).

Four representative mid-row trees were selected to monitor each cropping condition, the rest serving as border trees.

### 3.3. Measurements

Precipitation, air temperature, relative humidity, wind speed, and solar radiation were recorded in real-time by an automatic weather station located at the experimental site (http://www.cebas.csic.es/general_spain/est_meteo.html). Additionally, a combined air temperature and relative humidity sensor was installed at a height of 2 m in the middle of the shading net house. The vapour pressure deficit (VPD) for open-air and shade net were calculated hourly. The photosynthetically active radiation (PAR) was obtained from the measurements made by the quantum-sensor mounted in the chamber of the photosynthesis system (Li-6400, LI-COR, Lincoln, NE, USA).

A typical summer (9 August 2018) and winter (13 February 2019) day, representative of high and low atmospheric water demand conditions, respectively, served to evaluate plant water status. Discrete measurements of stem water potential (Ψ_stem_) and leaf gas exchange were made at 1 to 2 h intervals. Continuous records of canopy temperature (T_c_) were recorded.

The Ψ_stem_ was measured with a pressure chamber (Soil Moisture Equipment Corp., Model 3000, Santa Barbara, CA, USA) in mature leaves located near the trunk on the north face of the trees. One leaf per tree (four trees per cropping condition) was covered with aluminium foil at least 2 h before excision, following the recommendations described in [53].

Fully expanded (3 to 5 months old), sun-acclimated leaves (one leaf per tree and four trees per cropping condition, from the exterior canopy were used for gas exchange measurements: leaf net photosynthetic rate (P_n_), and stomatal conductance (g_s_) were measured with a portable photosynthesis system (LI-6400, Li-Cor, Lincoln, NE, USA) equipped with a broad-leaf chamber (6 cm^2^). Measurements were taken at near-constant ambient CO_2_ concentration (≈400 μmol mol^−1^) and the airflow rate inside the chamber was about 350 μmol s^−1^.

Canopy temperature (T_c_) was monitored using infrared radiometer sensors (Model SI-431 series, Apogee Instruments, Inc., Logan, UT, USA). The sensors were mounted on galvanized metal poles and installed 0.5 m above the tree canopy on two representative trees for each cropping condition. The half field of view (FOV) of these sensors was 14°, covering an area of 488 cm^2^ of the tree canopy. The IRT sensors were calibrated by the manufacturer. They were connected to the wireless sensor network, reading the values every 5 min and recording average values every 15 min.

The average values of T_c_ were computed and T_c_ − T_a_ values were calculated for open-air and shade conditions. The following index is proposed, based on 15-min lapsed T_c_ − T_a_ values. The canopy thermal index (CTI) was formulated as:

(1) $$CTI=\frac{(T_{c}-T_{a})-{\left(T_{c}-T_{a}\right)}_{min}}{{\left(T_{c}-T_{a}\right)}_{max}-{\left(T_{c}-T_{a}\right)}_{min}}$$

where, (T_c_ − T_a_)_min_: minimum T_c_ − T_a_ daily value and (T_c_ − T_a_)_max_: maximum daily T_c_ − T_a_ value. The index varies between 0 and 1.

### 3.4. Statistical Analysis

Data were analysed using IBM SPSS Statistic software (version 25, SPSS Inc., Chicago, IL, USA, 2002). Analysis of variance was performed and the mean values were compared by LSD0.05 test. Relationships between parameters were fitted to linear regressions.

## 4. Conclusions

The behaviour of plant water status indicators in lime trees and agro-meteorological variables were affected by both diurnal and seasonal changes and cropping conditions. The studied indicators identified different circadian rhythms, with maximum gas exchange and canopy temperature, and minimum stem water potential values, recorded at midday. Moreover, these plant indicators affected by seasonal variations had lower values in winter when the atmospheric demand conditions were also lower. The cropping conditions mainly affected the gas exchange of young lime tree leaves, the stomata of plants cultivated in the shaded conditions closing later and reaching maximum values after the plants cultivated in open-air. This would favour plant cooling, as indicated by the more negative values of the canopy-to-air temperature difference in summer. Then, shade netting is an interesting cropping technique and increasingly recognised as an alternative to open-air in semi-arid areas threatened by climate change, as the resulting increase in leaf gas exchange would favour carbon assimilation.

Due to the circadian rhythms of the plant water status indicators in leaves, we recommend that the stem water potential be determined at midday, coinciding with the maximum evaporative demand of the atmosphere and that gas exchange is measured early in the morning before the stomata of the lime leaves close.

The continuous measurement of canopy temperature using robust wireless infrared radiometers could serve as a good alternative to other classical physiological measurements for measuring plant water status. Although more research is needed on the use of ground-based thermal imagery to detect water stress threshold values in lime trees, the proposed canopy thermal index (CTI), virtually independent of the season of the year, could be used for tuning irrigation scheduling in areas where the scarcity of water resources demands precise irrigation.
